# Simultaneous Amelioratation of Colitis and Liver Injury in Mice by *Bifidobacterium longum* LC67 and *Lactobacillus plantarum* LC27

**DOI:** 10.1038/s41598-018-25775-0

**Published:** 2018-05-14

**Authors:** Se-Eun Jang, Jin-Ju Jeong, Jeon-Kyung Kim, Myung Joo Han, Dong-Hyun Kim

**Affiliations:** 1 Department of Life and Nanopharmaceutical Sciences, College of Pharmacy, Kyung Hee University, Seoul, 130-701 Korea; 2 Department of Food and Nutrition, Kyung Hee University, Seoul, 02447 Korea

## Abstract

Disturbances in the gut microbiota composition are associated with chronic inflammatory diseases of the intestine and the liver. In a preliminary study, *Lactobacillus plantarum* LC27 and *Bifidobacterium longum* LC67 could inhibit *Escherichia coli* growth and lipopolysaccharide-induced NF-κB activation linked to gut inflammation. Here, we investigated their effects on 2,4,6-trinitrobenzesulfonic acid (TNBS)-induced colitis and liver damage in mice. First, oral administration of LC27 or LC67 (1 × 10^9^ CFU/mouse) inhibited TNBS-induced colon shortening [F(5,30) = 100.66, *P* < 0.05] and myeloperoxidase activity [F(5,30) = 56.48, *P* < 0.05]. These probiotics restored TNBS-induced disturbance of gut microbiota, leading to the suppression of Proteobacteria to Bacteroidetes ratio and fecal and blood lipopolysaccharide levels. Second, LC27 and LC67 inhibited TNBS-induced NF-κB activation, reversed TNBS-suppressed tight junction protein expression, and restored Th17/Treg balance. Also, treatment with LC27 or LC67 significantly decreased TNBS-induced alanine transaminase [ALT, F(5,30) = 3.50, *P* < 0.05] and aspartate transaminase [AST, F(5,30) = 12.81, *P* < 0.05] levels in the blood, as well as *t*-butylhydroperoxide-induced ALT and AST levels. Finally, the mixture of LC27 and LC67 (0.5 × 10^9^ CFU/mouse, respectively) synergistically attenuated TNBS- or *t*-butylhydroperoxide-induced colitis and liver damage. The capability of LC27 and LC67 to reverse TNBS-mediated microbiota shift and damage signals suggests that these probiotics may synergistically attenuate colitis and liver injury by alleviating gut microbiota imbalance.

## Introduction

The gut microbiota consists of populations of bacteria, viruses, fungi, and protozoa in the gastrointestinal tract of hosts^1^. The composition of gut microbiota is affected by intrinsic and extrinsic factors, such as diet, drugs, hormones, and stress^2,3^. The majority of gut microbiota consists of bacteria, which play important roles in the health status of hosts^4^. For example, experimental colitis can be established in conventional laboratory animals, however, it does not significantly progress in germ-free animals. The gastrointestinal tract is the first organ affected by gut microbiota-generated byproducts such as endotoxins (lipopolysaccharide [LPS]), as well as and bacterial DNA and metabolites, followed by the liver^5,6^. The overexpression of LPS, as well as disturbances in the gut microbiota, can lead to inflammation in the gastrointestinal tract (e.g., colitis), and disruption in gut wall integrity and permeability; this further accelerates the absorption of byproducts, including endotoxins, into the liver. Excessive exposure to LPS also causes inflammation in the liver, and accelerates non-alcoholic and alcoholic liver diseases^5,7^. Dysregulated responses of the innate and adaptive immune systems against the gut microbiota are essential for the progression of colitis and liver injury in susceptible individuals^8,9^. The innate immune response is activated by many immune cells including macrophages^10^. These immune cells detect gut bacteria and their byproducts such as endotoxins, present their antigens to T cells, and induce adaptive immune responses^11^. Adaptive responses, such as activation of Th17 cells and regulatory T cells (Tregs), are thought to play major roles in the pathogenesis of colitis^12^. Th17 cells are promoted by IL-23, which is secreted by macrophages, and produce interleukin (IL)-17^13^. Th17 cells and IL-17 are involved in colitis^14^. Tregs suppresses Th17 cell proliferation and differentiation by regulating the expression of anti-inflammatory cytokines such as IL-10 and TGFβ^15,16^. Previous studies have shown that IL-10-deficient mice spontaneously develop colitis^17^. Therefore, immune modulators that regulate macrophage activation and Th17 cell and Treg differentiation via the gut microbiota-liver axis could simultaneously inhibit colitis and prevent liver injury.

Numerous studies have shown that functional foods including probiotics are beneficial for reducing the risks of metabolic and degenerative diseases and promoting good health^18,19^. Of these, lactobacilli and bifidobacteria have been reported to be beneficial microbes, as they support the maintenance of gut microbiota homeostasis in humans and animals^20,21^. These probiotics restore balance to gut microbiota composition^22^, induce host immune systems^23^, and have anti-obesity^24^, anti-hepatoprotective^25^, and anti-colitic effects^26^. *Bifidobacterium longum* alleviates dextran sulfate sodium (DSS)-induced colitis by suppressing IL-17A responses^27^. *Bifidobacterium infantis* inhibits colitis in mice by inducing Treg differentiation^28^. *Lactobacillus plantarum* C29 ameliorates age-dependent colitis in aged mice via inhibition of the NF-κB signaling pathways^29^. *Lactobacillus rhamnosus* GG also attenuates ethanol-induced liver injury in mice by restoring the gastrointestinal barrier via regulation of tight junction proteins and miR122a expression^30^. *Lactobacillus casei* MYL01 attenuates ethanol-induced liver damage *in vitro* by regulating the expression of proinflammatory cytokines such as tumor necrosis factor (TNF)-α and anti-inflammatory cytokine such as IL-10^31^. However, the effects of probiotics against both colitis and liver injury have not been thoroughly investigated.

Therefore, to understand the simultaneous effects of probiotics against colitis and liver injury, we screened for probiotics that can potently suppress bacterial growth and LPS production in *Escherichia coli*, and inhibited NF-κB activation in LPS-stimulated macrophages *in vitro*. Here, two probiotics, *Bifidobacterium longum* LC67 isolated from human fecal microbiota, and *Lactobacillus plantarum* LC27 isolated from kimchi, were selected to investigate their effects against 2,4,6-trinitrobenzenesulfonic acid (TNBS)-induced colitis and liver injury in mice.

## Results

### Effects of LC67 and LC27 on growth and LPS production of *E*. *coli*, and the innate and adaptive immune response *in vitro*

**Figure 1:**
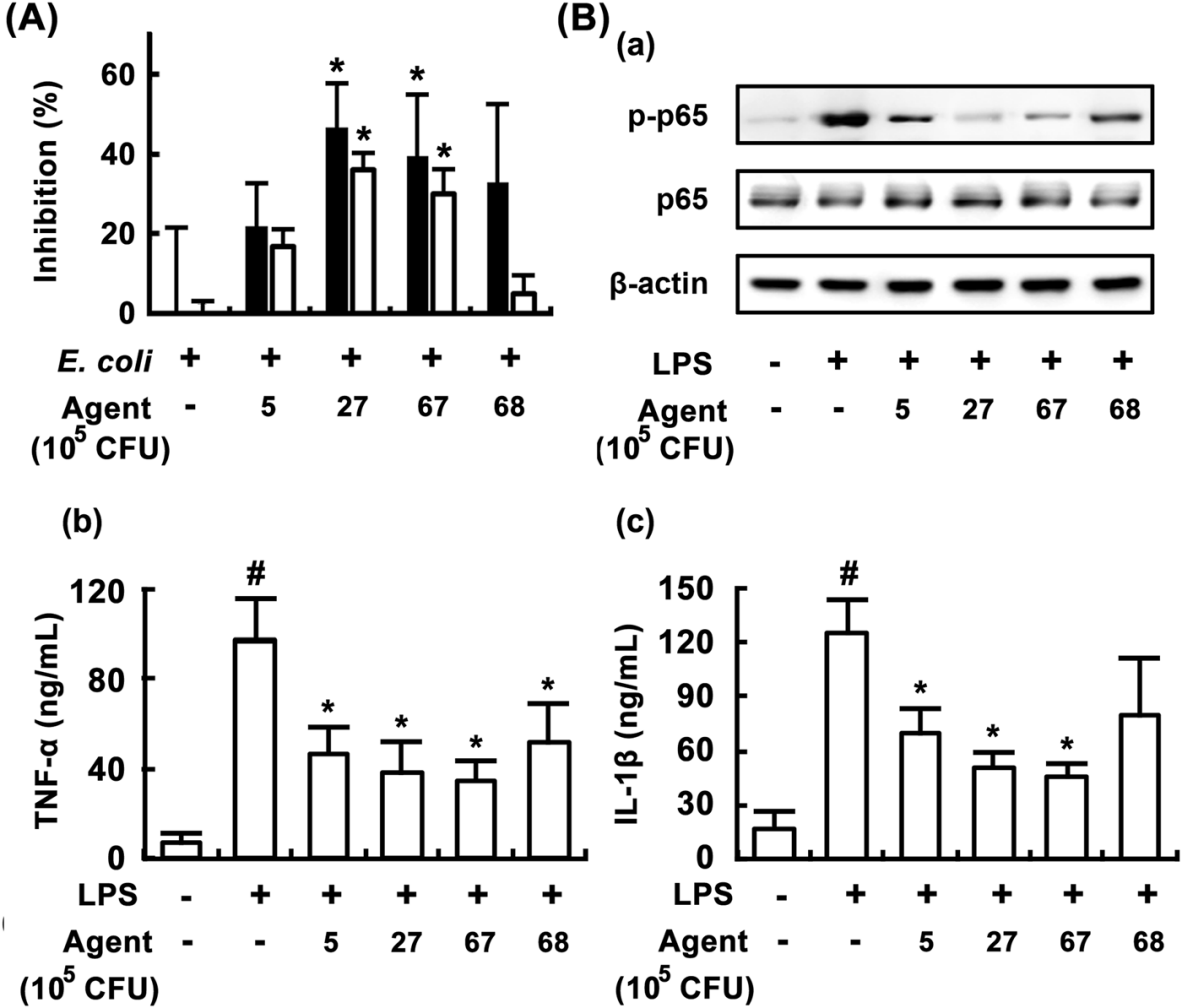
Inhibitory effects of bifidobacteria and lactobacilli isolated from human fecal microbiota and kimchi on the LPS production and growth of *Escherichia coli* and the NF-κB activation in LPS-stimulated macrophages. (**A**) Effects on bifidobacteria and lactobacilli on LPS production and growth of *E*. *coli*. *Lactobacillus brevis* L5, *L*. *plantarum* LC27, *Bifidobacterium longum* LC67, and *B*. *longum* LC68 (1 × 10^6^ CFU/mL) was anaerobically cultured in the presence of *E*. *coli* (1 × 10^6^ CFU/mL) in GAM (10 mL) and measured the number of *E*. *coli* growth (black bar) and level of LPS (white bar). The number of *E*. *coli* alone cultured for 24 h was 3.8 × 10^9^ CFU/mL and its LPS level was 8.2 ng/mL. (**B**) Effects on LPS-stimulated NF-κB (a) and TNF-α (b) and IL-1β expression (c). Peritoneal macrophages (0.5 × 10^6^ cells) were treated with 100 ng/mL of LPS in the absence or presence of probiotics (1 × 10^3^ or 1 × 10^5^ CFU/well) for 90 min (for NF-κB) or 24 h (for TNF-α and IL-1β). All data are shown as the mean ± SD (n = 4). ^#^*p* < 0.05 vs. normal control group. *p < 0.05 vs. group treated with LPS alone.

In order to screen lactobacilli and bifidobacteria that were capable of inhibiting growth and LPS production of *E*. *coli*, we isolated probiotics strains from the human gut microbiota and kimchi. Of the 100 isolated lactobacilli and bifidobacteria, LC27 and LC67 most potently exhibited the growth [F(4,15) = 4.12, *P* < 0.05] and LPS production [F(4,15) = 30.23, *P* < 0.05), followed by LC68 and LC5 (Fig. 1A). The other isolated bacteria did not inhibit them (Supplement Fig. S1). LC5, LC27, LC67, and LC68 also significantly inhibited NF-κB activation as well as TNF-α [F(5,18) = 13.43, *P* < 0.05] and IL-1β expression [F(5,18) = 11.36, *P* < 0.05] in LPS-stimulated macrophages (Fig. 1B). However, no cytotoxic effects against peritoneal macrophages under the experimental conditions were observed (data not shown).

**Figure 2:**
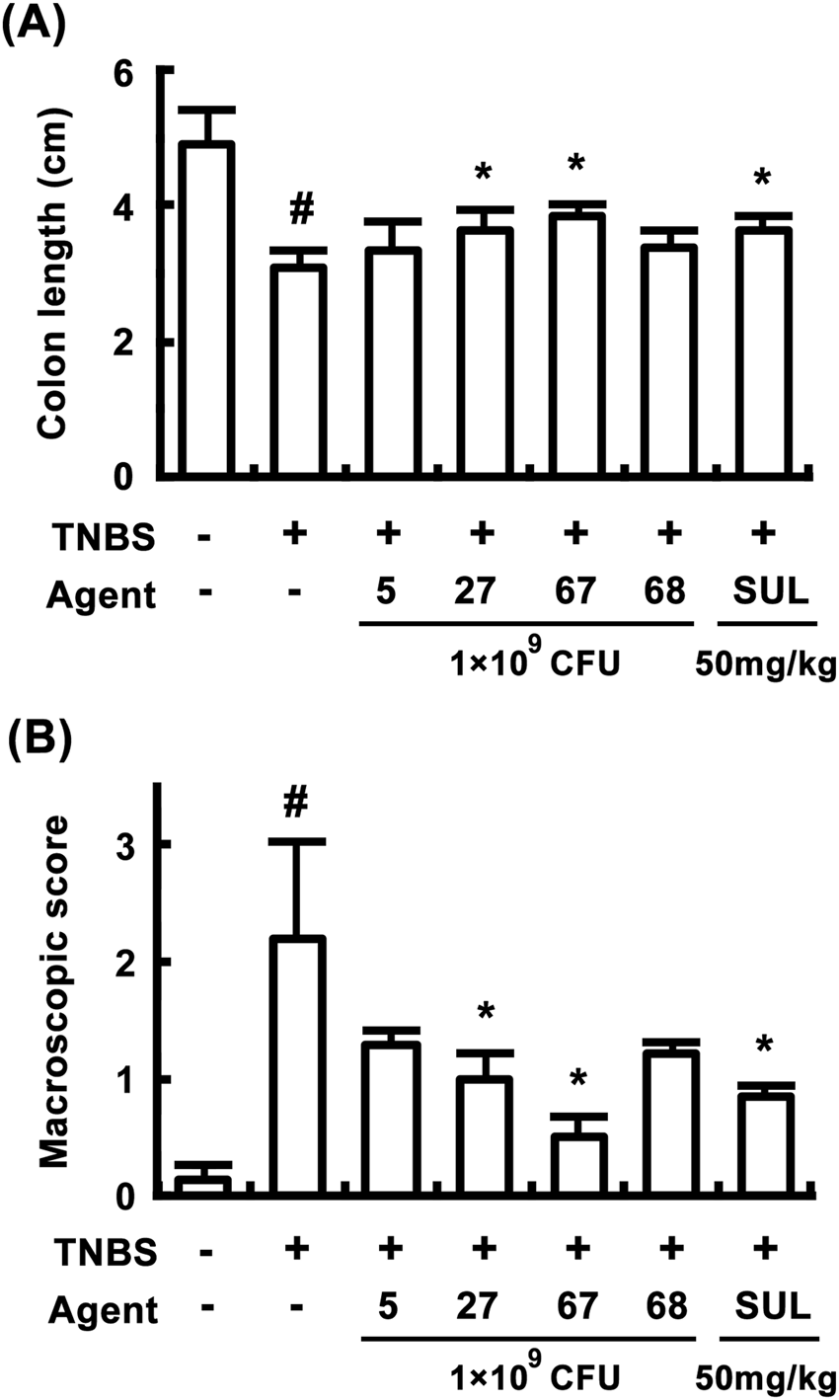
Anti-colitic effects of bifidobacteria and lactobacilli in mice. (**A**) Effects on colon length. (**B**) Effects on macroscopic score. TNBS, except in the normal control group, was intrarectally administered to mice and test agents [saline, LC5, LC27, LC67, LC68 (2 × 10^9^ CFU/mouse), or sulfasalazine (SUL; 50 mg/kg)] were orally administered for 3 days. All data are shown as the mean ± SD (n = 6). ^#^*p* < 0.05 vs. normal control group. *p < 0.05 vs. group treated with TNBS alone.

Next, we evaluated the anti-colitic effects of these probiotics in mice with TNBS-induced colitis (Fig. 2). Of these, LC27 and LC67 most potently inhibited TNBS-induced colitis markers, such as reduction in colon length [F(6,35) = 21.36, *P* < 0.05] and macroscopic score [F(6,35) = 21.54, *P* < 0.05]. LC27 and LC67 were identified as *Lactobacillus plantarum* and *Bifidobacterium longum*, respectively, based on results of Gram staining, sugar utilization testing (API 50 CHL Kit), and 16 S rRNA sequencing (ABI 3730XL DNA analysis).

### Anti-inflammatory effects of LC27 and LC67 in mice with TNBS-induced colitis

**Figure 3:**
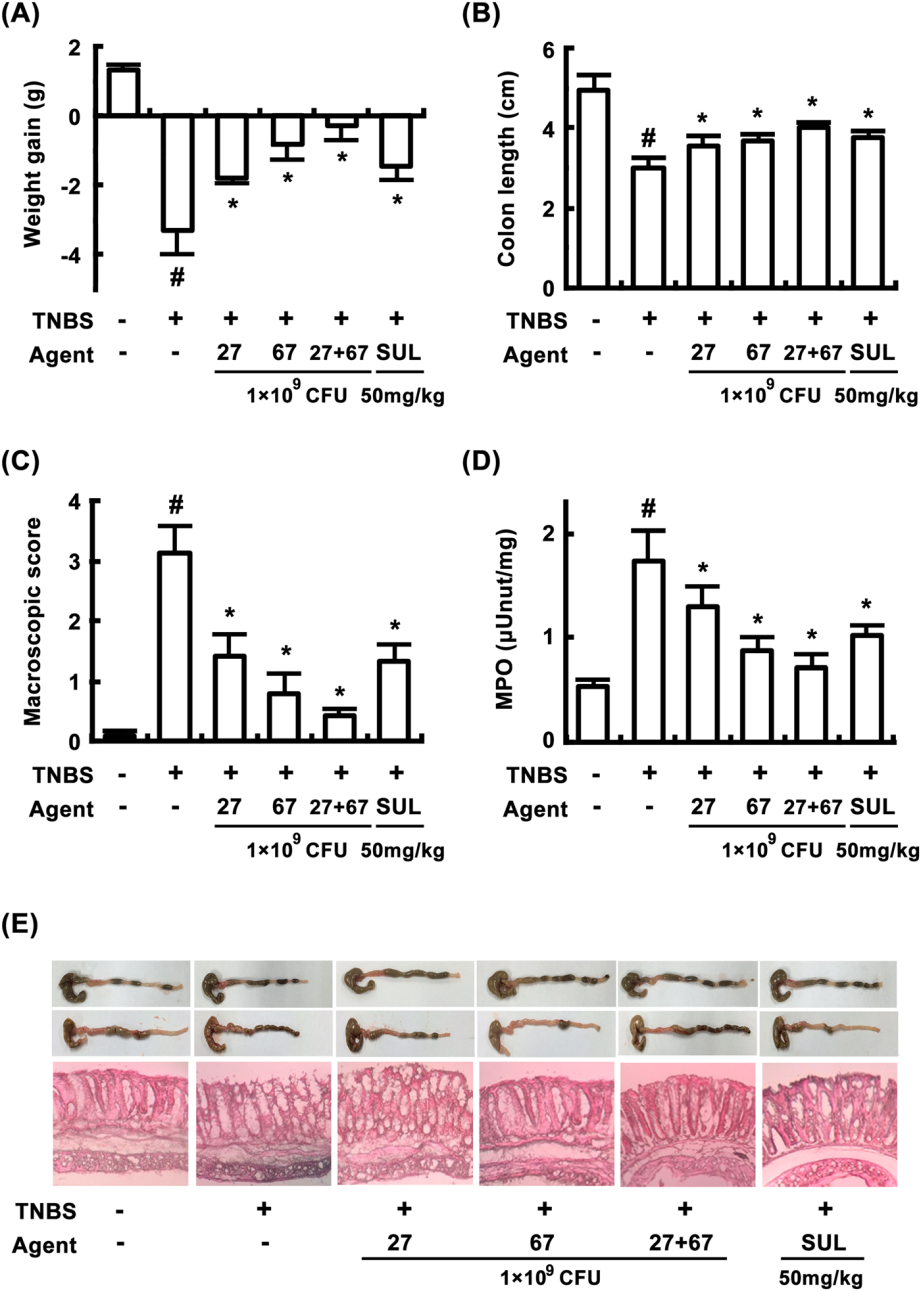
Effects of LC27, LC67, and PM on body weight (**A**), colon length (**B**), macroscopic disease (**C**), colonic myeloperoxidase (MPO) activity (**D**), and histological examination (**E**) in the colon of mice with TNBS-induced colitis. TNBS, except in the normal control group, was intrarectally administered to mice and test agents [saline, LC5, LC27, LC67, LC68 (2 × 10^9^ CFU/mouse), or sulfasalazine (SUL; 50 mg/kg)] were orally administered for 3 days. The mice were sacrificed 18 h after the final administration of test agents. All data are shown as the mean ± SD (n = 6). ^#^*p* < 0.05 vs. normal control group. *p < 0.05 vs. group treated with TNBS alone.

**Figure 4:**
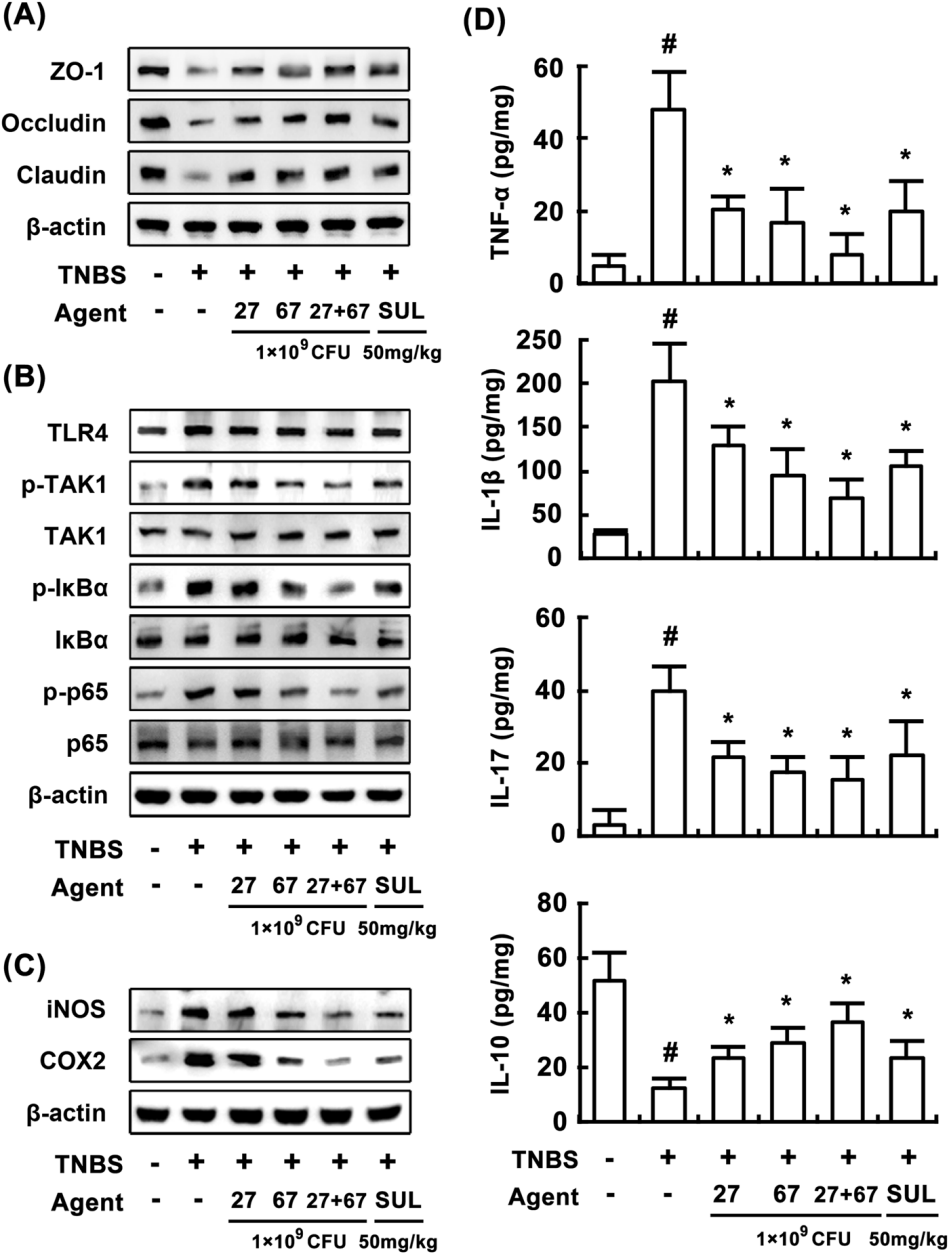
Effects of LC27, LC67, and PM on the expression of tight junction proteins (**A**), activation of NF-κB (**B**), iNOS and COX-2 (**C**), and expression of inflammatory cytokines (**D**) in mice with TNBS-induced colitis. TNBS, except in the normal control group, was intrarectally administered to mice and test agents [saline, LC5, LC27, LC67, LC68 (2 × 10^9^ CFU/mouse), or sulfasalazine (SUL; 50 mg/kg)] were orally administered for 3 days. Cytokines were determined by ELISA. iNOS, COX-2, and NF-κB signaling molecules were determined by immunoblotting. All values are shown as the mean ± SD (n = 6). ^#^*p* < 0.05 vs. normal control group. *p < 0.05 vs. group treated with TNBS alone.

In order to understand the anti-colitic properties of LC27 and LC67, we investigated the anti-inflammatory effects of LC27 and LC67 in mice with TNBS-induced colitis. TNBS caused severe inflammation in the colon, and resulted in colon shortening, increased myeloperoxidase activity, edema, and epithelial cell disruption by ulcerations (Fig. 3). Treatment with LC27 or LC67 (1 × 10^9^ CFU/mouse) inhibited TNBS-induced colon shortening [F(5,30) = 100.66, *P* < 0.05] and myeloperoxidase activity [F(5,30) = 56.48, *P* < 0.05] and suppressed edema and epithelial cell disruption in the colon (Fig. 3B,D,E). TNBS treatment also suppressed the expression of the colonic tight junction proteins such as claudin-1, occludin, and zonula occludens (ZO)-1 (Fig. 4A), whereas treatment with probiotics restored expression of tight junction proteins. Treatment with LC27 or LC67 inhibited TNBS-induced NF-κB activation, TAK1 and IκBα phosphorylation, as well as COX-2 and iNOS expression (Fig. 4B,C). In addition, they inhibited TNBS-induced expression of IL-1β [F(5,30) = 28.31, *P* < 0.05], IL-17 [F(5,30) = 21.84, *P* < 0.05], and TNF-α [F(5,30) = 29.49, *P* < 0.05], and increased TNBS-suppressed IL-10 expression [F(5,30) = 28.47, P < 0.05] (Fig. 4D).

To understand whether the LC27 and LC67 mixture has synergistic or antagonistic effects against colitis *in vivo*, we treated mice with LC27 (0.5 × 10^9^ CFU/mouse) and LC67 (0.5 × 10^9^ CFU/mouse) in combination, and compared the result to that of mice treated with LC27 or LC67 alone. Treatment with LC27 and LC67 mixture (PM) synergistically attenuated TNBS-induced colon shortening [F(5,30) = 100.66, *P* < 0.05] and myeloperoxidase activity [F(5,30) = 56.48, *P* < 0.05], and increased expression of tight junction proteins. LC27 and LC67 also synergistically inhibited NF-κB activation and reduced expressions of iNOS, COX-2, TNF-α, and IL-1β.

**Figure 5:**
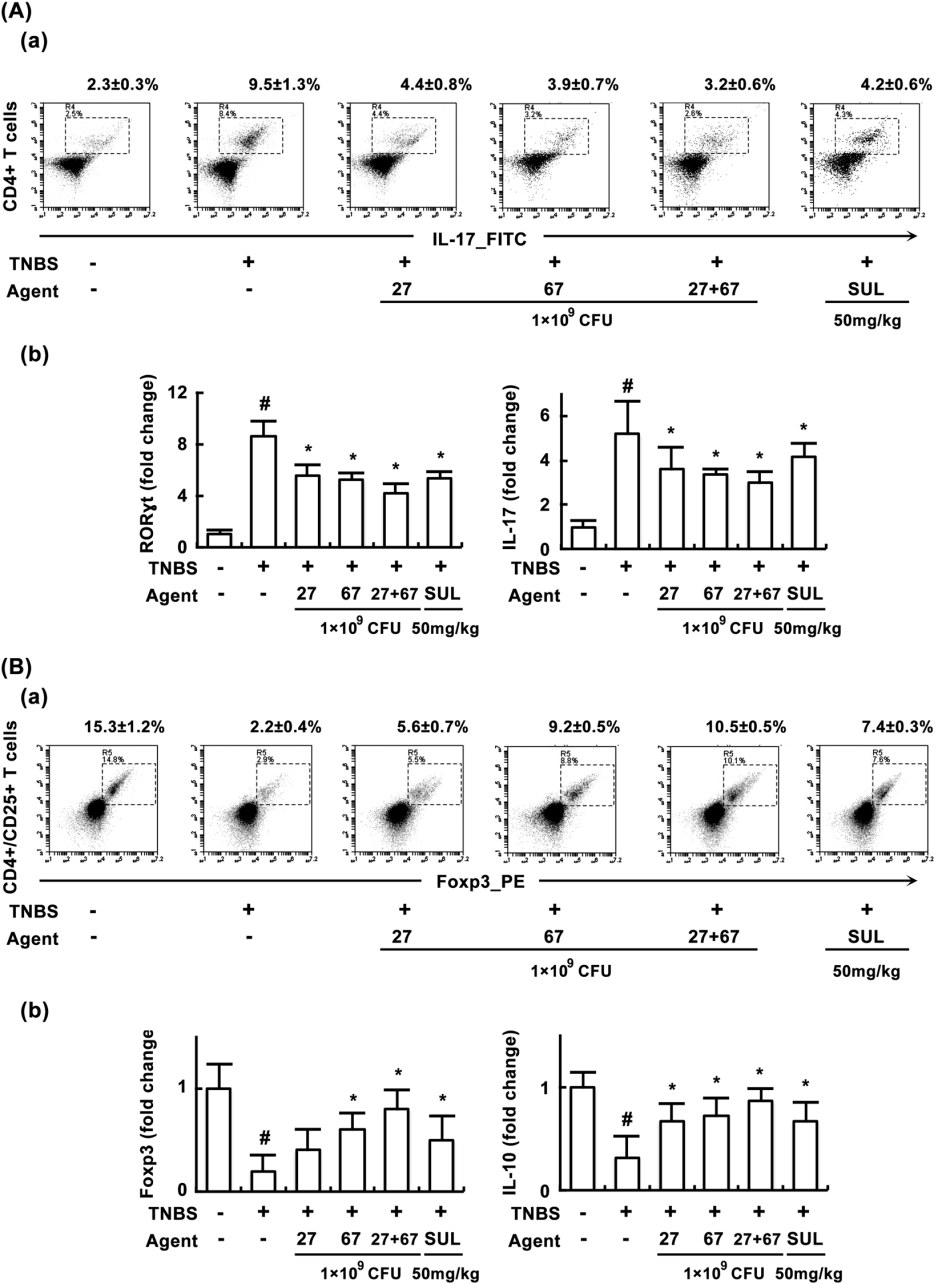
Effects of LC27, LC67, and PM on Th17 and Treg differentiation in the colon of mice with TNBS-induced colitis. (**A**) Effects on Th17 cell differentiation. (a) Effects on Th17 cell differentiation assessed by FACS. (b) Effects on ROTγt and IL-17 expression assessed by qRT-PCR. Cells isolated from the lamina propria were stained for cell surface CD4 and intracellular IL-17 and analyzed by flow cytometry. (**B**) Effects on Th17 cell differentiation. (a) Effects on Th17 cell differentiation assessed by FACS. (b) Effects on Foxp3 and IL-10 expression assessed by qRT-PCR. Cells isolated from the lamina propria were stained for the cell surface CD4 and CD25 and intracellular Foxp3 and analyzed by flow cytometry. TNBS, except in the normal control group, was intrarectally administered to mice and test agents [saline, LC5, LC27, LC67, LC68 (2 × 10^9^ CFU/mouse), or sulfasalazine (SUL; 50 mg/kg)] were orally administered for 3 days. All data are shown as the mean ± SD (*n = *6). All values are shown as the mean ± SD. (n = 6). ^#^*p* < 0.05 vs. normal control group. *p < 0.05 vs. group treated with TNBS alone.

In order to understand the effects of probiotics on the Th cell differentiation, we examined the effect of LC27 and LC67 on the differentiation of Th17 cells and Tregs in mice with TNBS-induced colitis (Fig. 5A,B). Treatment with TNBS significantly increased and decreased Th17 and Treg differentiation in the lamina propria of colons, respectively. Treatment with LC27 or LC67 inhibited TNBS-induced Th17 cell differentiation (Fig. 5A), and increased Treg cell differentiation (Fig. 5B). qPCR anlaysis revealed that TNBS significantly upregulated RORγt and IL-17 expression, and suppressed Foxp3 and IL-10 expression. However, treatment with LC27 or LC67 inhibited the TNBS-induced expression of RORγt [F(5,30) = 124.83, *P* < 0.05] and IL-17 [F(5,30) = 19.10, *P* < 0.05], and increased the TNBS-suppressed expression of Foxp3 [F(5,30) = 12.32, *P* < 0.05] and IL-10 [F(5,30) = 11.64, P < 0.05]. As expected, PM down-regulated TNBS-induced Th17 cell differentiation and RORγt and IL-17 expressions, and upregulated Treg cell differentiation and FoxP3 and IL-10 expressions.

### Ameliorating effects of LC27, LC67, and PM against TNBS-induced liver injury in mice

**Figure 6:**
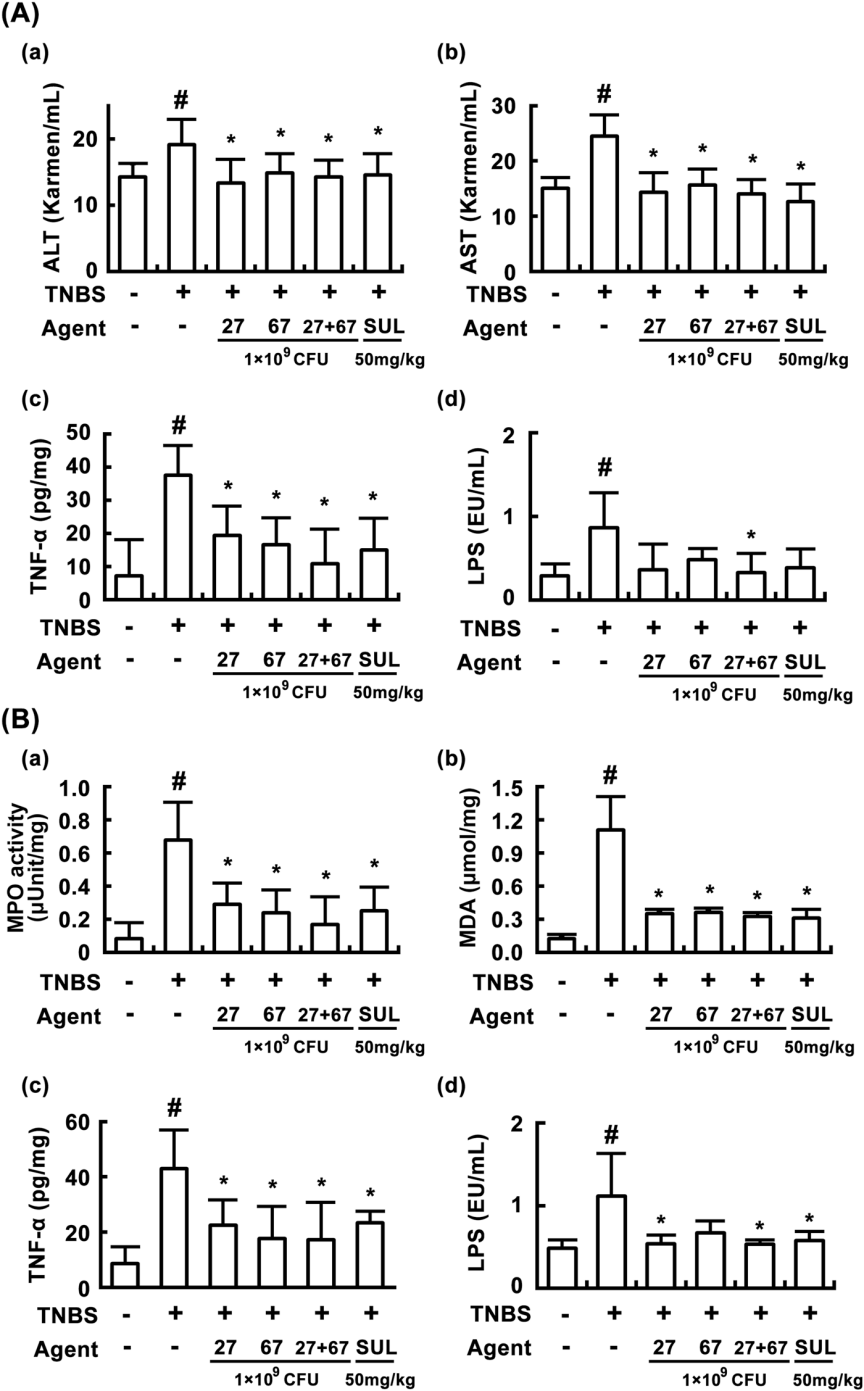
Effects of LC27, LC67, and PM on TNBS-induced liver injury in mice. (**A**) Effects on blood ALT (a), AST (b), TNF-α (c), and LPS levels (d). (**B**) Effects on liver myeloperoxidase (MPO) (a), malondialdehyde (MDA) (b), TNF-α (c), and LPS levels (d). TNBS, except in the normal control group, was intrarectally administered to mice and test agents [saline, LC5, LC27, LC67, LC68 (2 × 10^9^ CFU/mouse), or sulfasalazine (SUL; 50 mg/kg)] were orally administered for 3 days. LPS was determined using LAL assay kit. All values are shown as the mean ± SD (n = 6). ^#^*p* < 0.05 vs. normal control group. *p < 0.05 vs. group treated with TNBS alone.

**Figure 7:**
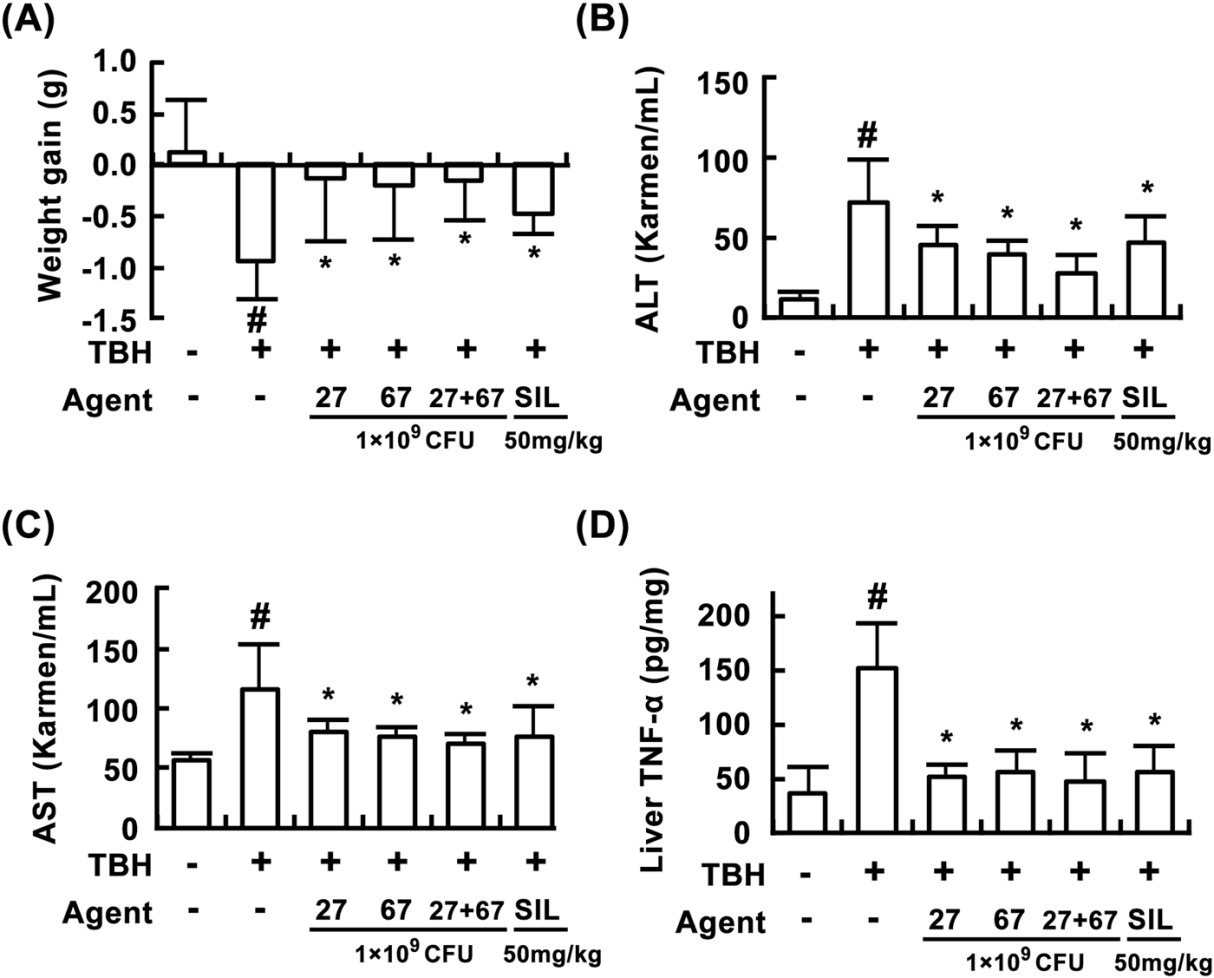
Effects of LC27, LC67, and PM on *t*-BHP-induced liver injury in mice. Effects on body weight (**A**), blood ALT (**B**), AST (**C**), and liver TNF-α levels (**D**). *t*-BHP, except in the normal control group, was intraperitoneally injected to mice and test agents [saline, LC27 (2 × 10^9^ CFU/mouse), LC67 (2 × 10^9^ CFU/mouse), PM (the mixture of LC27 and LC67 [1:1]; 1 × 10^9^ CFU/mouse), or silymarin (SIL, 50 mg/kg)] were orally administered for 3 days. All values are shown as the mean ± SD (n = 6). ^#^*p* < 0.05 vs. normal control group. *p < 0.05 vs. group treated with TNBS alone.

In order to investigate whether TNBS treatment could induce liver damage in mice, we measured biomarkers for the liver damage in mice with TNBS-induced colitis. TNBS treatment increased levels of alanine transaminase (ALT) and aspartate transaminase (AST) in the blood, as well as levels of TNF-α in the blood and the liver: it caused liver damage (Fig. 6A). TNBS treatment also increased myeloperoxidase activity and MDA level in the liver (Fig. 6B). However, oral administration of LC27, LC67, or PM for 3 days significantly decreased TNBS-induced ALT [F(5,30) = 3.50, *P* < 0.05], AST [F(5,30) = 12.81, *P* < 0.05], TNF-α [F(5,30) = 8.45, *P* < 0.05], and LPS level [F(5,30) = 4.29, *P* < 0.05], in the blood, as well as myeloperoxidase activity and MDA and TNF-α levels in the liver (Fig. 6A,B). Additionally, these probiotics attenuated body weight (F = 4.07, *P* < 0.05), blood ALT (F = 10.97, *P* < 0.05), AST (F = 6.45, *P* < 0.05), and liver TNF-α levels (F = 16.15, *P* < 0.05) in mice with *tert*-butyl hydroxyperoxide (*t*-BHP)-induced liver injury (Fig. 7).

### Effects of LC27, LC67, and PM on TNBS-induced gut microbiota disturbance in mice

**Figure 8:**
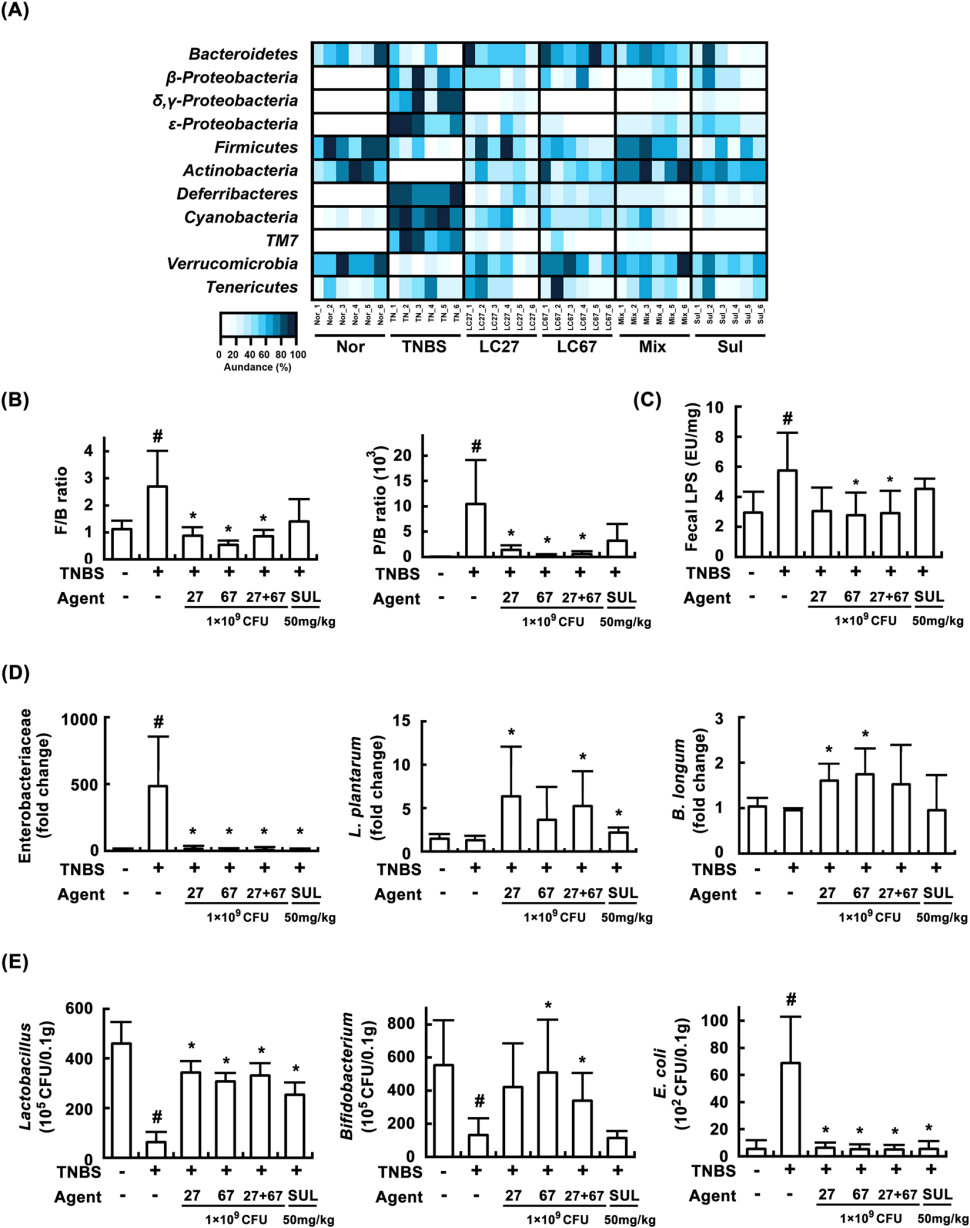
Effects of LC27, LC67, and PM on gut microbiota composition in mice with TNBS-induced colitis and liver injury. (**A**) Effects on gut microbiota, assessed by qPCR. (**B**) Effects on the ratio of *Firmicutes* to *Bacteroidetes* (F/B) and *Proteobacteria* to *Bacteroidetes* ratio (P/B). (**C**) Effects on gut microbiota LPS levels, assessed by LAL assay kit. (**D**) Effects on the populations of Enterobacteriaceae, *Lactobacillus plantarum*, and *Bifidobacterium longum*, assessed by qPCR. (**E**) Effects on the levels of *Lactobacillus* sp., *Bifidobacterium* sp., *E*. *coli*, assessed by the culture of selective media. Test agents and saline were orally administered for 3 days after TNBS treatment. TNBS, except in the normal control group, was intrarectally administered to mice and test agents [saline, LC27, LC67, PM (1 × 10^9^ CFU/mouse), or sulfasalazine (SS; 50 mg/kg)] were orally administered for 3 days. All values are shown as mean ± SD (n = 6). ^#^*p* < 0.05 vs. normal control group. *p < 0.05 vs. group treated with TNBS alone.

Previous studies have shown that TNBS treatment increases the ratio of Firmicutes/Bacteroidetes, and elevates LPS production in the gut microbiota^32^. Moreover, the overexpression of LPS in the gut microbiota leads to gastrointestinal inflammation^24^ and excessive exposure to LPS causes inflammation in the liver^5,7^. Therefore, we investigated the effects of LC27, LC67, and PM on the gut microbiota composition and LPS production in mice with TNBS-induced colitis and liver injury (Fig. 8). TNBS treatment increased the ratio of Firmicutes/Bacteroidetes, like previously reported^32^. Furthermore, TNBS treatment increased the number of Proteobacteria but reduced the number of Bacteroidetes, resulting in a reduced Proteobacteria/Bacteroidetes ratio [F(5,30) = 5.22, *P* < 0.05] (Fig. 8B). Treatment with LC27, LC67, or PM significantly inhibited the TNBS-induced Proteobacteria level, and increased the TNBS-suppressed Bacteroidetes level (Fig. 8A). Moreover, TNBS treatment significantly increased the LPS level [F(5,30) = 3.85, *P* < 0.05] in the colonic fluid and blood of mice, whereas LC27, LC67, or their mixture significantly decreased the TNBS-induced LPS production (Fig. 8C). TNBS treatment also increased Enterobacteriaceae levels including *Escherichia coli*. However, these probiotics reversed the suppression of lactobacilli [F(5,30) = 31.36, P < 0.05] and bifidobacteria [F(5,30) = 4.08, P < 0.05] by TNBS, and reduced the amount of Enterobacteriaceae and *Escherichia coli* [F(5,30) = 18.34, *P* < 0.05], which belong to phylum Proteobacteria (Fig. 8B,C). Furthermore, these treatments increased the populations of *Lactobacillus plantarum* and *Bifidobacterium longum*. Collectively, these data suggest that LC27 and LC67 can alleviate TNBS-induced gut microbiota imbalance.

## Discussion

Acute and chronic inflammations are the body’s response to injuries and infections^33,34^. Acute inflammation is a normal and beneficial response to injury, whereas chronic inflammation is persistent and excessive. Inflammatory reactions in the gastrointestinal tract can be activated by a variety of stresses such as excessive ROS, alcohol, and LPS owing to disturbance of gut microbiota^34^. Exposure to alcohol or high fat diet causes dysbiosis via alteration in the gut microbiota, including an increase in Proteobacteria and a decrease in Bacteroidetes^5^. An increase in the Gram-negative Proteobacteria reduces the expression of cellular tight junctions and increases gut permeability through overexpression of LPS, resulting in increased absorption of LPS into the blood. LPS, a major driver of systemic inflammation^35^, increases blood TNF-α levels via Toll-like receptor 4-associated NF-κB signaling pathway to cause inflammation, even though blood TNF-α level is barely detectable in mice in absence of any stimuli or treatment^36^. Therefore, chronic inflammatory responses lead to progressive damage to the body, resulting in a variety of chronic inflammatory diseases, such as colitis, hepatitis, and rheumatoid arthritis^37^. To regulate these inflammatory diseases, probiotics may be used to control TNF-α expression via regulation of NF-κB in immune cells, as well as to inhibit LPS production induced by the gut microbiota imbalance.

In the present study, we found that LC27 and LC67 inhibited TNBS-induced colitis via inhibition of NF-κB in macrophages and epithelial cells, similar to previous reports^38^. Furthermore, treatment with these probiotics restored TNBS-disturbed gut microbiota composition: they suppressed the population of Enterobacteriaceae, particularly *Escherichia coli*, which is belonging to Proteobacteria, and gut microbiota LPS levels and increased the populations of lactobacilli and bifidobacteria, including *Lactobacillus plantarum* and *Bifidobacterium longum*. Additionally, it has been shown that *Lactobacillus brevis* G-101 inhibits TNF-α and IL-1β expression in macrophages, leading to attenuation of colitis^39^. In addition, *Bifidobacterium longum* CH57 was found to attenuate colitis by inhibiting NF-κB signaling pathways and TNF-α expression. *Lactobacillus plantarum* C29 also ameliorates colitis in aged mice by inhibiting NF-κB signaling^29^. Another study has demonstrated that *Lactobacillus casei* DN-114001 inhibits DSS-induced colitis by inhibiting gut membrane permeability and NF-κB activation^40^. Furthermore, some probiotics were also shown to restore composition of gut microbiota and fecal LPS level in mice with colitis^29,40^. These results suggest that probiotics can inhibit NF-κB activation and restore disrupted gut microbiota composition to attenuate colitis. Furthermore, treatment with these probiotics significantly suppressed blood LPS and TNF-α levels in mice with TNBS-induced colitis. These treatments also reduced liver MDA and myeloperoxidase activity, and blood AST and ALT levels, resulting in attenuation of TNBS-induced liver injury in mice. In spite of short-term treatment with these probiotics, their effects were comparable to those of sulfasalazine, a positive agent. Another study showed that *L*. *rhamnosus* CCFM1107 reduces oxidative stress and restores intestinal flora in ethanol-treated mice, which ameliorates liver injury^41^. In addition, *L*. *rhamnosus* GG reestablishes the gastrointestinal barrier via the suppression of tight junction proteins and miR122a in mice, leading to the alleviation of ethanol-induced liver injury^30^. Lastly, *L*. *acidophilus* CSG exhibited hepatoprotective effects in mice during CCl_4_ and *t*-BHP-induced oxidative stress by restoring the disturbed gut microbiota^26^. These results suggest that probiotics such as LC27 and LC67 are effective against colitis, and could attenuate oxidative stress-induced liver injury and colitis by inhibiting NF-κB activation, scavenging ROS, and alleviating gut microbiota imbalance.

Activated macrophages secrete IL-23, which induces expression of proinflammatory cytokines such as TNF-α and IL-6, and also promotes the differentiation and activation of Th17 cells^10,33,34^. Th17 cells suppress Treg the differentiation, resulting in the onset of chronic inflammatory diseases such as colitis^13^. Conversely, activated Tregs inhibit Th17 cell the differentiation via secretion of IL-10 and TGF-β, which results in attenuation of chronic colitis. Therefore, the Th17/Treg cell balance is important for the development of colitis.

In the present study, we found that LC27 and LC67 inhibited Th17 cell differentiation and RORγt expression, and enhanced Treg differentiation and Foxp3 expression, leading to attenuation of colitis and liver injury. Furthermore, an increase in IL-10 expression was also observed both *in vitro* and *in vivo*. It has been previously shown that *Lactobacillus brevis* CH23 restores Th17/Treg balance via regulation of the transcription factors Foxp3 and RORγt, as well as the cytokines IL-17 and IL-10, resulting in recovery from colitis^28^. Similarly, *Lactobacillus casei* MYL01 attenuates ethanol-induced liver damage *in vitro* via regulation of TNF-α and IL-10 expression^31^. *Lactobacillus casei* also suppresses the development of rheumatoid arthritis by upregulating IL-10 expression^42^. *Bifidobacterium longum* ameliorates inflammatory diseases by suppressing IL-17 expression^27^. These results suggest that LC23 and LC67 may inhibit colitis and liver injury by correcting the imbalance of Th17/Treg cells involved in adaptive immunity, via regulation of innate immune cells and gut microbiota composition, and by increasing the expression of colonic tight junction proteins.

Although commercial probiotic products contain a combination of various probiotics such as Lactobacilli, Bifidobacteria, and Streptococci the combined effects of these probiotic mixtures have not been thoroughly investigated^20^. For example, *Bifidobacterium longum* CH57 and *Lactobacillus brevis* CH23 synergistically inhibit colitis by inhibiting macrophage activation and restoring Th17/Treg balance^26^. In the present study, the LC27 and LC67 mixture PM, synergistically rather than additively, attenuated TNBS-induced colitis such as colon shortening, myeloperoxidase activity, TNF-α and IL-10 expression, and Th17 and Treg cell differentiation. However, TNBS-induced liver damage was significantly attenuated by treatment with PM. This involved correcting the Th17/Treg imbalance via regulation of innate immune cells and gut microbiota composition and increasing the expression of colonic tight junction proteins. Furthermore, these probiotics also attenuated *t*-BHP-induced liver injury. These results suggest that synergistic probiotic products containing a combination of bacterial species, such as PM, may be more effective in protecting diseased caused by imbalance of the gut microbiota.

In conclusion, *Lactobacillus plantarum* LC27, isolated from kimchi, suppressed gut bacterial LPS production and NF-κB activation in macrophages. *Bifidobacterium longum* LC67, isolated from the human gut microbiota, inhibited gut bacterial LPS production and differentiation of splenic T cells into Th17 cells. It also increased Treg cell differentiation via up-regulation of IL-10 and Foxp3 expression. These probiotics synergistically attenuated TNBS-induced colitis and liver injury as well as *t*-BHP-induced liver injury by correcting the gut microbiota composition and inhibiting inflammatory responses involved in innate and adaptive immunity. Collectively, our study supports that LC27 and LC67 could be effective tools to control colitis and liver damage induced by altered gut microbiota landscape.

## Materials and Methods

### Materials

LPS purified from *Escherichia coli* O111:B4, TNBS, *t*-BHP, collagenase type VIII, RPMI 1640 were purchased from Sigma-Aldrich (St. Louis, MO, U.S.A.). Antibodies for COX-2, ERK, p-ERK, IκBα, p- IκBα, IRAK1, p-IRAK1, iNOS, p65, p-p65, TAK1 and p-TAK1 were purchased from Cell Signaling Technology (Beverly, MA, U.S.A.). Radioimmuno-precipitation assay (RIPA) buffer, tetramethyl benzidine was purchased from Sigma (St Louis, MO, U.S.A.). ELISA kits for IL-1β, IL-6, IL-10, and TNF-α were purchased from R&D Systems (Minneapolis, MN, U.S.A.). mRNA isolation kit was purchased from Qiagen (Hilden, Germany). A diazo-coupled limulus amoebocyte lysate (LAL) assay kit was purchased from Cape Cod Inc. (E. Falmouth, MA, USA). Pan T Cell Isolation Kit II was purchased from MiltenyiBiotec GmbH (Bergisch Gladbach, Germany). Anti-CD28, anti-CD3, recombinant IL-6, and recombinant TGF-β were purchased from BioGems International Inc. (Westlake Village, CA, U.S.A.). Fetal bovine serum (FBS) and heat-inactivated fetal calf serum (FCS) purchased from Panbiotech GmbH (Aidenbach, Germany). de Man, Rogosa and Sharpe (MRS) medium for probiotics was purchased from BD (Sparks, MD, USA). General anaerobic medium (GAM) for probiotics and other bacteria were purchased from Nissui Pharmaceutical Co (Tokyo, Japan). Other chemicals used were of the highest grade available.

### Probiotics preparation

Probiotics including *Lactobacillus plantarum* LC27 and *Bifidobacterium longum* LC67 were cultured in general media for probiotics. Briefly, these probiotics were grown to the density of 2 - 4 × 10^9^ CFU/mL and centrifuged to harvest cells. The collected cells (1 × 10^10^ CFU/mL) were suspended in phosphate buffered saline (inactivated at 80 °C for 30 min, used for *in vitro* experiments) or 1% glucose (for *in vivo* experiments).

### Animals

Male C57BL/6 (21–23 g, 6-weeks old) were supplied from RaonBio Inc. (Seoul, Korea). All animals were housed in wire cages at 20–22 °C and 50 ± 10% humidity, fed standard laboratory chow and water ad libitum. After the acclimation for 7 days, mice were used in experiments.

All animal experiments were approved by the Committee for the Care and Use of Laboratory Animals in the Kyung Hee University and performed in accordance with the Kyung Hee University Guidelines for Laboratory Animals Care and Usage (IRB No., KHUASP(SE)-16-049).

### Preparation of macrophages

Macrophages were prepared according to the method of Jeong *et al*.^29^. Mice were intraperitoneally injected with 4% (w/v) thioglycolate solution (2 mL) and killed 4 days after the injection^29^. Cells were removed with RPMI 1640 in the peritoneal cavity, centrifuged (300 × g, 10 min), and washed with RPMI 1640 twice. Collected cells (1.5 × 10^6^ cells/well) were incubated in RAF at 37 °C for 20 h and washed three times. The attached cells were used as macrophages. To evaluate the anti-inflammatory effect of probiotics, macrophages (1 × 10^6^ cells/well) were treated with LPS (100 ng/mL) in the absence or presence of each probiotic (1 × 10^3^ or 1 × 10^5^ CFU/mL) for 90 min (for p65 and p-p65) or 24 h (for TNF-α and IL-1β).

### Preparation of mice with experimental colitis and liver injury

First, mice were randomly divided into 7 groups: normal control, TNBS-induced colitic control groups treated with vehicle, four probiotics (1 × 10^9^ CFU), or sulfasalazine (50 mg/kg). Each group consisted of six mice.

Second, mice were randomly divided into 6 groups: normal control, TNBS-induced colitic control groups treated with vehicle, LC27 (1 × 10^9^ CFU/mouse), LC67 (2 × 10^9^ CFU/mouse), and their mixture (1:1, each 1 × 10^9^ CFU/mouse), or sulfasalazine (50 mg/kg). Each group consisted of six mice. Colitis was induced by the intrarectal injection of 2.5% (w/v) TNBS solution (100 μL, dissolved in 50% ethanol) into the colon of mice anesthetized with ether^43^. Normal control group was treated with vehicle alone instead of TNBS. To entirely distribute TNBS within the colon, mice were held in a vertical position for 30 s after the TNBS injection. Test agents (probiotics or sulfasalazine dissolved in 1% glucose) were orally administered once a day for 3 days after TNBS treatment. Mice were killed 18 h after the final administration of test agents. Normal control group was treated with vehicle alone instead of test agents. Whole-blood samples were immediately withdrawn from carotid artery. Sera were prepared by centrifugation (10 min, 250 × g) and ALT, AST, and TNF-α levels were then determined according to the method of Lee *et al*.^44^. The colon was removed and opened longitudinally. The colitis grade was macroscopically scored (0, no ulcer and no inflammation; 1, no ulceration and local hyperemia; 2, ulceration with hyperemia; 3, ulceration and inflammation at one site only; 4, two or more sites of ulceration and inflammation; 5, ulceration extending more than 2 cm). The colons were gently washed by ice-cold phosphate buffered saline (PBS) and were stored at −80 °C until used in the experiment for, myeloperoxidase activity assay, ELISA, and immunoblotting.

### Preparation of mice with t-BHP-induced hepatic injury

Mice with *t*-BHP-induced hepatic injury were prepared according to the method of Lee *et al*.^44^. Firs, mice were randomly divided into 6 groups: normal control, *t*-BHP-treated control groups treated with vehicle, probiotics (LC27, LC67, or their mixture (1:1): 1 × 10^9^ CFU/mouse), or silymarin (50 mg/kg). Each group consisted of six mice. Mice were intraperitoneally treated with 1.5 mmol *t*-BHP/kg. Test agents were orally administered once a day for 3 days from 24 h after treatment with *t*-BHP. Control group was given with saline instead of the sample compounds. Blood samples were collected 18 h after the final administration of test agents by cardiac puncture under ether anesthesia. Sera were obtained by centrifugation (1000 × g, 15 min) and ALT, AST, and TNF-α levels were then determined according to the method of Lee *et al*.^44^.

### Histological examination

Colons or livers were fixed in 50 mM phosphate buffer (pH 7.4) containing 4% paraformaldehyde overnight, frozen in optimal cutting temperature solution, cut into 15 μm section using a cryostat, stained with hematoxylin-eosin, and then observed under a light microscopy^43^.

### Assay of myeloperoxidase (MPO) activity

Mouse colon or liver tissues were homogenized in 10 mM potassium phosphate buffer (pH 7.0) containing 0.5% hexadecyl trimethyl ammonium bromide, and centrifuged for 10 min at 20,000 × g at 4 °C^44^. The resulting supernatants (50 μL) were added to the reaction mixture containing 0.1 mM H_2_O_2_ and 1.6 mM tetramethyl benzidine preincubated at 37 °C for 2 min, and sequentially monitored the absorbance (650 nm) at 37 °C for 5 min. Myeloperoxidase activity was calculated as the quantity of enzyme degrading 1 μmol/mL of peroxide, and expressed in unit/mg protein protein^43^. The amount of protein was determined by the method of Bradford.

### Quantitative real time - polymerase chain reaction (qPCR)

Reverse transcription was performed with 2 μg of total RNA isolated from the colon. Real time PCR for IL-10, IL-17, Foxp3, RAR-related orphan receptor gamma t (RORγt), and glyceraldehyde 3-phosphate dehydrogenase (GAPDH) was performed as described previously^43^, utilizing Qiagen thermal cycler, which used SYBER premix agents. Thermal cycling conditions were as follows: activation of DNA polymerase at 95 °C for 5 min, followed by 36 cycles of denaturation and amplification at 95 °C for 5 s and 63 °C for 30 s, respectively. The normalized expression of the assayed genes, with respect to GAPDH, was computed for all samples by using the Microsoft Excel data spreadsheet. Primers were used as follows^43^: IL-10 forward, 5′-ATG CTG CCT GCT CTT ACT GAC TG-3′, reverse, 5′-CCC AAG TAA CCC TTA AAG TCC TGC-3′; IL-17 forward, 5′-TTT AAC TCC CTT GGC GCA AAA-3′ reverse, 5′-CTT TCC CTC CGC ATT GAC AC-3′; RORγt forward, 5′-ACAGCCACTGCATTCCCA GTTT-3′, reverse, 5′- TCTCGGAAGGACTTGCAGACAT-3′; Foxp3 forsward, 5′-CCC ATC CCC AGG AGT CTT-3′, reverse, 5′-ACC ATG ACT AGG GGC ACT GTA-3′; and GAPDH forward, 5′-TGC AGT GGC AAA GTG GAG AT-3′, reverse, 5′-TTT GCC GTG AGT GGA GTC AT-3′.

Real time PCR for Firmicutes, Proteobacteria, Actinobacteria, and Bacteroidetes was performed with 100 ng of total DNA isolated from the colon fluid, utilizing Takara thermal cycler, which used SYBER premix agents^45^. Thermal cycling conditions were as follows: activation of DNA polymerase for 30 s at 95 °C, followed by 35 cycles of denaturation and amplification at 95 °C for 5 s and 63 °C for 30 s, respectively. The normalized expression of the assayed genes, with respect to bacterial rRNA, was computed for all samples using the Microsoft Excel data spreadsheet. Primers were used as follows^45,46^: Firmicutes forward, 5′-GGA GYA TGT GGT TTA ATT CGA AGC A-3′, reverse, 5′-AGC TGA CGA CAA CCA TGC AC-3′; Bacteroidetes forward, 5′-GTT TAA TTC GAT GAT ACG CGA G-3′ reverse, 5′-TTA ASC CGA CAC CTC ACG G-3′; Actinobacteria forward, 5′-TGT AGC GGT GGA ATG CGC-3′, reverse, 5′-AAT TAA GCC ACA TGC TCC GCT-3′; δ/γ-Proteobacteria forward, 5′-GCT AAC GCA TTA AGT RYC CCG-3′, reverse 5′-GCC ATG CRG CAC CTG TCT-3′; Enterobacteriaceae forward, 5-TGC CGT AAC TTC GGG AGA AGG CA-3′, reverse, 5′-TCA AGG ACC AGT GTT CAG TGT C-3′; *Bifidobacterium longum* forward, 5′-CAG TTG ATC GCA TGG TCT T-3′, reverse, 5′-TAC CCG TCG AAG CCA C-3′; *Lactobacillus plantarum* forward, 5′-TCA TGA TTT ACA TTT GAG TG-3′, reverse, 5′-GAC CAT GCG GTC CAA GTT GTT-3′; and bacterial 16S rRNA forward, 5′-AGA GTT TGA TCC TGG CTC AG-3′, reverse 5′-AAG GAG GTG WTC CAR CC-3′.

### Determination of LPS

The content of LPS was determined using a LAL assay kit according to manufacturer’s protocol^29^. For the assay of culture medial LPS contents, each probiotic (1 × 10^6^ CFU/mL) and *E*. *coli* (1 × 10^6^ CFU/mL) was simultaneously inoculated in GAM (5 mL) and anaerobically cultured for 37 °C for 24 h. The cultured suspension was sonicated for 1 h on ice, centrifuged at 5,000 × *g* for 10 min, filtrated through a 0.45 μm filter followed by re-filtration through a 0.22 μm filter, and inactivated at 70 °C for 10 min.

For the assay of fecal LPS contents, colon content from mice (100 mg) were placed in 50 mL of PBS in a pyrogen-free tube and sonicated for 1 h on ice. After centrifugation at 400 × *g* for 10 min, the supernatant was collected, sterilized by filtration through a 0.45 μm filter followed by re-filtration through a 0.22 μm filter, and inactivated at 70 °C for 10 min.

For the assay of blood LPS contents, serum (5 μL) was diluted 1:10 in pyrogen-free water, inactivated for 10 min at 70 °C, and incubated with LAL solution for 30 min at 37 °C.

Each filterate or serum (50 μl) was incubated with LAL solution at 37 °C for 30 min, added additional reagents to formation of a magenta derivative, and measured the absorbance at 545 nm.

### Flow cytometry of Th17 and Treg cells in the lamina propria of colons

For the assay of Th17 cells and Tregs, colons were cut into small pieces, incubated with 2.5 mM EDTA at 37 °C with agitation for 20 min, minced, and digested for 20 min with RPMI containing 1 mg/mL collagenase type VIII at 37 °C. Lamina propria cells were then prepared^44^. T cells were isolated using a Pan T cell Isolation Kit II, fixed and stained with anti-FoxP3 or anti-IL-17A antibodies, and then analyzed by flow cytometry (C6 Flow Cytometer® System, San Jose, CA, USA).

### ELISA and immunoblotting

Colon, liver tissues, and cultured cells were homogenized in the RIPA lysis buffer (1 mL) containing 1% phosphatase inhibitor cocktail and 1% protease inhibitor cocktail at 4 °C and centrifuged at 15,000 × g for 15 min.

For the determination of cytokines, the supernatants of tissue homogenates and cultured cells were transferred to a 96-well microplate. IL-1β, IL-10, IL-17, and TNF-α expression levels were determined using ELISA kits^44^.

For the immunoblotting, the supernatants of tissue and cultured cell homogenates were subjected to electrophoresis on sodium dodecyl sulfate-polyacrylamide gel, transferred to nitrocellulose membrane, blocked with non-fat dried-milk proteins, probed with antibodies, and washed with PBS with tween 20^43^. Proteins were detected with horseradish peroxidase-conjugated secondary antibodies. Protein bands were visualized with an enhanced chemiluminescence detection kit.

### Statistical analysis

All data are indicated as the mean ± standard deviation (SD), with statistical significance analyzed using one-way ANOVA followed by Duncan’s multiple range test (*P* < 0.05).

## Electronic supplementary material

Supplementary Information
