# Supplementary material for: Simultaneous Amelioratation of Colitis and Liver Injury in Mice by Bifidobacterium longum LC67 and Lactobacillus plantarum LC27
Source: Sci Rep. 2018 May 14;8:7500. doi: 10.1038/s41598-018-25775-0 (PMC5951891; doi:10.1038/s41598-018-25775-0)
Supplement: Supplementary file 1 — Supplementary Information [file 41598_2018_25775_MOESM1_ESM.docx]

**[Supplementary Information]**

**Simultaneous Amelioratation of Colitis and Liver Injury in Mice by *Bifidobacterium longum* LC67 and *Lactobacillus plantarum* LC27**

Se-Eun Jang^a,b^, Jin-Ju Jeong^a^, Jeon-Kyung Kim^a^, Myung Joo Han^b^, and Dong-Hyun Kim^a,*^

*^a^Department of Life and Nanopharmaceutical Sciences, College of Pharmacy, Kyung Hee University, Seoul 130-701, Korea; ^b^Department of Food and Nutrition, Kyung Hee University, Seoul, 02447, Korea*

**Isolation of probiotics**

Chinese cabbage kimchi (10 g) was suspended in MRS broth (100 mL) and the supernatant was serially diluted with MRS broth, inoculated in MRS agar plates, and anaerobically incubated at 37^o^C for 2 days and 50 colonies with gram-positive bacilli were selected according to the method of Kim et al.^1^ Fresh feces of twenties humans (approximately 2 g) was collected, suspended in GAM broth (50 mL), centrifuged at 500 *g* for 5 min, and the supernatants were serially diluted with GAM broth, inoculated in BL agar plates, and anaerobically incubated at 37^o^C for 2 days and 50 colonies with gram-positive bacilli were selected according to the method of Jang et al.^2^

**Selection of probiotics inhibiting *Escherichia coli* growth and LPS production**

*Escherichia coli* (1 × 10^6^ CFU/mL), which was previously cultured in TS broth at 37^o^C for 24 h, and each isolated probiotic (1 × 10^6^ CFU/mL), which was previously cultured in GAM broth, were anaerobically cocultured in GAM broth (10 mL) at 37^o^C for 24 h, then inoculated in DHL agar plates, and *E. coli*-positive colonies were counted. Additionally, the coculture was collected at 10,000 *g* and sonicated for 1 h on ice. After centrifugation at 400 *g* for 10 min, the supernatant was filtered through a 0.45-μm filter followed by re-filtration through a 0.22-μm filter. The LPS level of the supernatant was then measured using a LAL Assay Kit.^3^

The selected probiotics were identified using the Gram staining, sugar utilization test (API 50 CHL Kit, bioMerieux, Seoul, Korea), and 16S rRNA sequencing using an ABI 3730XL DNA analyzer (Thermo Fisher Scientific Inc., Waltham, MA, USA).


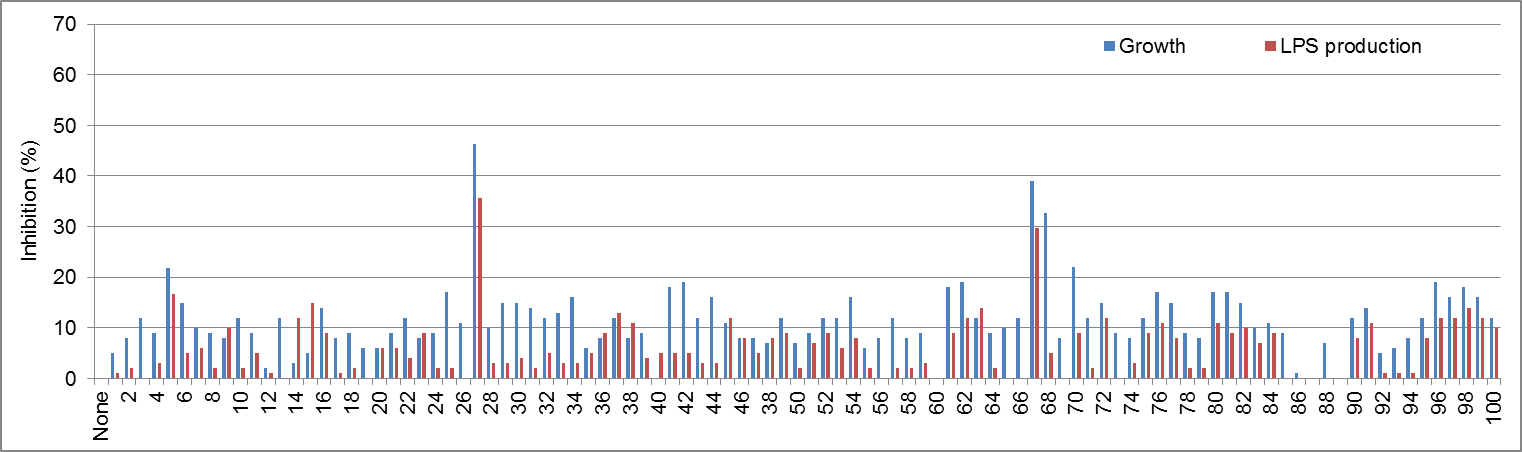


**Figure S1.** Inhibitory effects of probiotics isolated from kimchi (No. 1 - 50) and human fecal microbiota (No. 51 - 100) on the LPS production and growth of *Escherichia coli* and the NF-κB activation in LPS-stimulated macrophages. Each isolated probiotic candidate (1×10^6^ CFU/mL) was anaerobically cultured in the presence of *E. coli* (1 × 10^6^ CFU/mL) in GAM (10 mL) and measured the number of *E. coli* growth and level of LPS.

**References**

1. Jeong, J.J., Kim, K.A., Jang, S.E., Woo, J.Y., Han, M.J., & Kim, D.H. Orally administrated *Lactobacillus pentosus* var. *plantarum* C29 ameliorates age-dependent colitis by inhibiting the nuclear factor-kappa B signaling pathway via the regulation of lipopolysaccharide production by gut microbiota. *PLoS One*, 10, e0116533 (2015).

2. Jang, H.M., Jang, S.E., Han, M.J., & Kim, D.H. Anxiolytic-like effect of Bifidobacterium adolescentis IM38 in mice with or without immobilisation stress. Benef Microbes 9, 123-132 (2018).

3. Kim, K.A., Jeong, J.J., & Kim, D.H. *Lactobacillus brevis* OK56 ameliorates high-fat diet-induced obesity in mice by inhibiting NF-κB activation and gut microbial LPS production. *J Funct Foods* 13, 183-191 (2015).
